# “I Can Only Work So Hard Before I Burn Out.” A Time Sensitive Conceptual Integration of Ideological Psychological Contract Breach, Work Effort, and Burnout

**DOI:** 10.3389/fpsyg.2018.00131

**Published:** 2018-02-09

**Authors:** Samantha K. Jones, Yannick Griep

**Affiliations:** ^1^Department of Psychology, University of Calgary, Calgary, AB, Canada; ^2^Division of Epidemiology, Stress Research Institute, Stockholm University, Stockholm, Sweden

**Keywords:** ideological psychological contracts, work effort, burnout, threshold, dynamics

## Abstract

Employees often draw meaning from personal experiences and contributions in their work, particularly when engaging in organizational activities that align with their personal identity or values. However, recent empirical findings have demonstrated how meaningful work can also have a negative effect on employee’s well-being as employees feel so invested in their work, they push themselves beyond their limits resulting in strain and susceptibility to burnout. We develop a framework to understand this “double edged” role of meaningful work by drawing from ideological psychological contracts (iPCs), which are characterized by employees and their employer who are working to contribute to a shared ideology or set of values. Limited iPC research has demonstrated employees may actually work harder in response to an iPC breach. In light of these counterintuitive findings, we propose the following conceptual model to theoretically connect our understanding of iPCs, perceptions of breach, increases in work effort, and the potential “dark side” of repeated occurrences of iPC breach. We argue that time plays a central role in the unfolding process of employees’ reactions to iPC breach over time. Further, we propose how perceptions of iPC breach relate to strain and, eventually, burnout. This model contributes to our understanding of the role of time in iPC development and maintenance, expands our exploration of ideology in the PC literature, and provides a framework to understanding why certain occupations are more susceptible to instances of strain and burnout. This framework has the potential to guide future employment interventions in ideology-infused organizations to help mitigate negative employee outcomes.

## Introduction

Employees are driven to pursue meaningful work, to contribute to a greater cause or set of values, and to feel engaged and fulfilled. Meaningful work tends to be positively associated with satisfaction, well-being (Hackman and Oldham, 1980; Arnold et al., 2007; Blustein, 2008), and organizational commitment (Tyler and Blader, 2003), and is negatively associated with perceptions of negative working conditions and turnover intentions (Arnoux-Nicolas et al., 2016). Unfortunately, meaningful work can also act as a “double-edged sword” where employees are willing to make personal sacrifices to ensure that the key objectives of their occupation are maintained (Bunderson and Thompson, 2009). This “double-edge sword” of meaningful work can be better understood when focusing on the shared ideology-infused obligations between the organization and the employee—labeled ideological psychological contracts (iPCs; Thompson and Bunderson, 2003)—that often make up these meaningful work arrangements. Specifically, a shared focus on ideology creates meaningful work that may simultaneously be beneficial and detrimental to employees because of the challenges associated with the shared value’s ability to positively enhance employees’ personal identity and negatively contribute to employees’ well-being by pushing them to exceed their personal limits to ensure the ideology is maintained.

The PC—defined as a continuous exchange of a set of reciprocal obligations, arising from explicit and implicit promises, between the employee and the employer (Rousseau, 2001)—and perceptions of PC breach—the cognitive evaluation that delivered inducements do not equate to what was obligated (Morrison and Robinson, 1997)—form an ideal framework to understand this “double-edge sword” of meaningful work. Although substantial empirical progress has been made in understanding the relationship between PC breach and employee attitudes and behaviors (for meta-analyses, see Zhao et al., 2007; Bal et al., 2008), little attention has been given to employee reactions that do not fit our pre-existing beliefs about the employee reactions to perceptions of PC breach. That is, we tend to rely on the negative norm of reciprocity where an employee reciprocates negative treatment with a similarly negative behavior or attitude (Gouldner, 1960) and meta-analytical findings (Zhao et al., 2007; Bal et al., 2008) to argue that PC breach incontrovertibly leads to a decrease in performance. However, in doing so, we are ignoring important recent research findings that do not align with this “decreased performance” finding and hint toward important contextual factors. Most essential in this respect are recent findings by Vantilborgh et al. (2014) that demonstrate that some PC breaches may actually result in increased work effort rather than decreased performance. The authors argue that this finding can potentially be explained by the employees’ (volunteers in the study of Vantilborgh et al., 2014) desire to protect a shared valued cause or purpose, even in the aftermath of the organization’s perceived failure to fulfill the PC. Indeed, based on Social Identity Theory (Tajfel and Turner, 1979, 1985), this shared valued cause or purpose is central to employees because it represents a shared identity between employees and their employer that warrants protection when threatened. Hence, in lieu of sacrificing their investment in the valued cause or purpose, we argue that these employees will continue to push themselves and increase their work effort to compensate for the perceived failure of their organization, even when this might come at a personal cost (e.g., increased strain and burnout; Demerouti et al., 2001).

**FIGURE 1:**
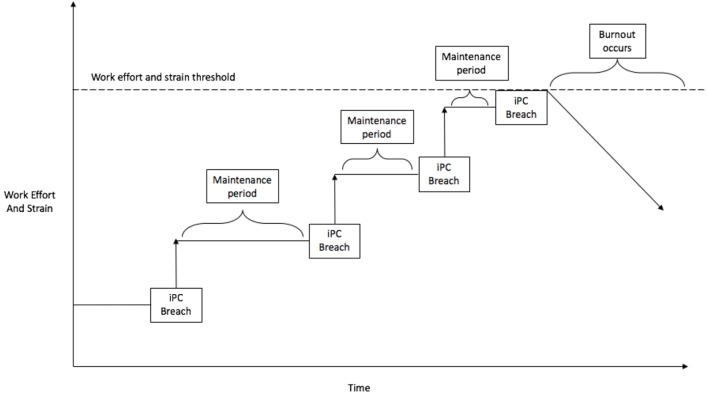
Ideological psychological contract (iPC) breach in relation to employee’s increased work effort, strain, and burnout over time. Initial magnitude of work effort and strain are relative.

The purpose of this conceptual paper is to propose how perceptions of iPC breach in relation to employee’s increased work effort evolve over time, and how this process might eventually lead to the experience of strain and burnout. An illustration of this relationship can be found in **Figure 1**. We posit that the central importance of a valued cause or purpose in an iPC might shape how employees engage in sense-making after having perceived an iPC breach. We aim to advance the larger PC literature by framing the evaluation of iPC breach as a threshold where not all deviations from an organization’s obligation are perceived as an iPC breach but rather that certain events may fall within a personal zone of acceptance and hence do not trigger an employee reaction (Schalk and Roe, 2007; Rigotti, 2009). However, when the deviation exceeds one’s personal threshold, we argue that employees will increase their work effort to compensate for the organization’s failure to contribute to the shared valued cause or purpose. However, we argue that increasing one’s work effort in the aftermath of experiencing an iPC breach comes at the cost of developing strain (Gakovic and Tetrick, 2003). In addition, we argue that this process is not a static one, but rather that as the number of iPC breaches increases over time, this personal zone of acceptance become more narrow (i.e., lowered threshold), resulting in more deviations exceeding the tolerance limit and further increased perceptions of iPC breach and felt strain. Finally, we argue that this process will eventually reach a breaking point at which employees are no longer able to continue to increase their work effort in the aftermath of an iPC breach, and the pent-up strain will eventually translate in burnout.

We first review the literature on iPCs. Next, we develop our conceptual model and make propositions about the mechanisms by which iPC breach accumulates over time and has the potential to lead to burnout. We discuss the role this framework plays in extending both the theoretical iPC and burnout literature through a temporal lens and how this framework can practically help ideology-focused industries address burnout among their employees. Finally, we make explicit methodological recommendation to explore our conceptual model.

### Ideological Psychological Contracts

Ideological psychological contracts represent the shared mutual agreements between employees and their organization that are built on a set of shared values, mission, and/or purpose the organization in believed to strive for (Thompson and Bunderson, 2003). iPCs are often considered to be held in conjunction with other types of PCs (relational and transactional) and contribute to the overall norms of reciprocity expected within a mutual employee–employer exchange relationship. Employees enter into these PCs with the understanding that the organization is committed to, and can legitimately support, activities that support the ideology (e.g., ensuring enough resources/supplies in a healthcare facility, developing policies and procedures that support environmentally friendly behavior) and in return, employees contribute their time, skills, and energy to carry out activities related to the cause (e.g., providing high-quality patient care, recycling in their office).

These PCs are unique from other relational (socio-emotional) and transactional (economical) PC types in that negotiation of obligations is focused on a larger shared ideology that is culturally or socially understood rather than a focus on the individual employee–organization interaction (Thompson and Bunderson, 2003). As a result, these relationships are built on the reciprocal exchange of ideological currency where both the employee and their organization are committed to support the cause in subjectively equal forms. In the case of the employee, this may be done by taking initiative to support the cause in a new way or engaging in extra-role behaviors. From the organizational perspective, ideological currency can be exchanged through public support of the cause and their employees, signaling a credible commitment to the ideology. Although the behaviors within the iPC may have relational and transactional elements (e.g., providing financial resources), the goals of the iPC are oriented toward contributing to the prosocial values and/or purpose of the organization.

Perceptions of breach among these PCs can also differ. That is, transactional PCs tend to have clearly defined organizational obligations and non-negotiable guidelines for fulfillment and non-fulfillment (e.g., paid fair compensation), whereas relational PCs allow for more negotiable perceptions of fulfillment (e.g., provided opportunities for career development), and iPCs have aspects of both. For example, iPCs can have flexible interpretations (negotiable) as to what entails fulfillment from the organizational and breach of a value driven obligation as well as actions that an organization may engage in that are considered “moral hot buttons” (non-negotiable; Thompson and Bunderson, 2003). Employees consider these organizational actions as inflexible and, regardless of past experiences, perceive them as an iPC breach. For example, if an organization with a strong human rights ideology began outsourcing labor to areas of the world that condone unethical work conditions and unfair wages, employees of that organization may perceive this as an iPC breach even when all other obligations of the PC are fulfilled.

Similar to relational and transactional PC breaches, employees may attribute the causes of the iPC breach to intentional actions by their organization (renegading) or to a misunderstanding of the agreement (incongruence; Thompson and Bunderson, 2003). However, Thompson and Bunderson (2003) also proposed two additional attributions employees may make when determining whether an iPC breach occurred. Employees may perceive that the goals of their organization have changed in favor of organizational and economic survival (perceived goal displacement) or that the ideology of the organization is threated due to partnerships with other organizations that do not share the same values (perceived goal interpenetration). Although all three types of PCs (i.e., transactional, relational, and ideological) may involve different employer and employee obligations and attributions for PC breach, they in combination guide the expectations within a mutual employee–employer exchange relationship. As such, employees may experience a PC breach of one PC type while maintaining fulfillment of organizational obligations in the other PC types. This may explain why employees can experience severe PC breaches in their relational and transactional PCs, while simultaneously continuing to pursue the ideology of their iPC (Thompson and Bunderson, 2003).

Moreover, because iPCs exceed the individual employee–employer interaction, the source of the perceived PC breach may also be different. That is, while transactional and relational PCs are focused largely on the direct violation of the employee that holds the PC (e.g., an employee does not receive the promotion that they were promised), iPC breaches may be the result of indirect disruptions and violations (e.g., a decrease in funding that supports patient care) (Thompson and Bunderson, 2003). As a corollary of this distinction, employees may perceive a breach of their iPC even if the organizational action was not directed at them (i.e., the decreased funding affected patient care, not employee salaries and benefits). This implies that, unlike relational and transactional PCs where employees are only monitoring the fulfillment of inducements that are directly related to their obligations, iPCs affect employees monitoring of much more diverse types organizational activities. In turn, this may affect the organizational representatives the employee holds accountable for fulfillments and breach of iPCs.

Based on the above theoretical arguments, the way iPCs are developed and navigated by employees is unique from relational and transactional PCs. The mutual agreements that form the iPC are highly related to employees’ work context and the value employees place on aspects of their personal and organizational ideology. As a result, what is perceived as an iPC breach for one employee, may not be perceived as an iPC by another employee. The long-term pursuit of ideology in the organization may be sufficient to buffer some employees from perceiving iPC breach but may prime others to find short-comings in their organizations effort (Thompson and Bunderson, 2003). Despite the theoretical exploration of what iPCs may look like, we currently have limited empirical understanding and support regarding how perceptions of iPCs come about, how these iPC breaches are related to one another over time, and how iPCs evolve and impact employment outcomes over time. The limited research that has begun to address these topics has largely focused on the formation of iPCs such as how the schemas of iPC relationships can be based on occupational norms and ideologies, particularly in specific professions such as knowledge workers (O’Donohue et al., 2007), doctors (Bunderson, 2001), nurses (O’Donohue and Nelson, 2007; McCabe and Sambrook, 2013), and zookeepers (Bunderson and Thompson, 2009). For example, O’Donohue et al. (2007) found that knowledge workers were more concerned with the ideological currency of their PCs rather than how their transactional or relational PCs were being navigated, and that organizational support of ideological concerns tied to productivity. Similarly, Bunderson (2001) examined how the competing professional and administrative ideologies of doctors could profoundly impact how they view their profession and how they react to PC breaches; PC breaches related to professional ideologies were more likely to be associated with lower organizational commitment and job performance whereas PC breaches related to administrative ideologies were more strongly associated with dissatisfaction, turnover intentions, and actual turnover. When considering managers in educational settings, Bal and Vink (2011) found that when organizations better fulfilled ideology based obligations (e.g., provided opportunities to contribute to society), employees felt a higher sense of obligation to continue contributing to the ideology of the organization. These findings support the notion that exchanges of ideological currency in organizations can drive the reciprocal relationship between employee and employer and contribute to the overall organizational mission and vision.

Overall, these findings illustrate how different expectations and experiences of shared values can influence how employees evaluate PC breach and the types of responses they may have in the aftermath of PC breach.

### Ideological Psychological Contract Breach and Time

Recently, the field of PC research has put forward a strong temporally focused research agenda (for a PC and time critique, see Hansen and Griep, 2016). Within this push for a more dynamic understanding of the processes underlying PC breach, we argue that far too little attention has been given to the role of time in understanding the unfolding nature of iPC breach perceptions and employee reactions. That is, to date most PC research has remained predominantly contemporaneous and has overlooked the temporal context in which perceptions of PC breach and employee reactions interact and potentially change in intensity over time. This is especially true for the more limited exploration of the iPC with the vast majority of the research relying on cross-sectional data or very limited time-points to explain a dynamic relationship (O’Donohue and Nelson, 2007; Bingham et al., 2014; Vantilborgh et al., 2014). As a result, we know very little about how iPCs behave over time and how multiple perceptions of iPC breach affect employee well-being and workplace outcomes. This gap is our knowledge is problematic largely because we know that PC breach is not an uncommon event in the workplace; the majority of employees have experienced a PC breach in their career (Robinson and Rousseau, 1994) and PC breaches even happen as often as on a weekly basis (Conway and Briner, 2002). While these studies have demonstrated a great deal about the frequency of PC breaches and negative affective reactions, we have yet to fully understand how perceptions of PC breaches accumulate over time and what this frequency means for other long-term organizational and employee outcomes. Further, we know that employees with iPCs strongly identify with the core value or purpose of their work, which warrants additional consideration of how perceptions of PC breach of ideological PCs unfold over time (Bunderson and Thompson, 2009). Without considering these keys aspects of PCs, we fail to adequately consider the true impact of PC breach in context.

To address the above discussed critique, we argue that employees’ experiences of iPC breach over time can be understood through the lens of Affective Events Theory (AET; Weiss and Cropanzano, 1996). AET, and supporting empirical findings (Zhao et al., 2007), temporally arrange workplace events (i.e., iPC breach) as preceding negative affective reactions (i.e., strain) which in turn, result in changes to workplace attitudes and behaviors (i.e., work effort and burnout). In line with AET, we argue that workplace events, such as an iPC breach, are conceptualized as a threat to the employee’s principle goal of supporting the valued cause or purpose. According to AET, an incongruence between the importance of this valued cause for employees and resources or support they receive from their organization to achieve this valued cause (e.g., funding to support patient care), may result in strong negative affective reactions (i.e., strain), which in turn further contribute to changes in workplace attitudes and behaviors.

Extending this temporal lens beyond isolated event-reaction relationships, AET (Weiss and Cropanzano, 1996) can also be used to understand the effect of multiple iPC breach perceptions over prolonged periods of time. Weiss and Cropanzano (1996) integrate the concept of affective cycles to explain how workplace events can disrupt baseline affect in employees. That is, over time, employees follow cycles of moods and emotions that are disrupted by novel events, causing a spike in negative affect, which then return to a starting baseline. However, as this relationship continues over time, the ebb and flow of the affective cycles may change. In the context of iPC breach, repeated disruptions in affective cycles may contribute to how employees anticipate future workplace events. That is, employees do not perceive iPC breach with a blank slate but rather situate these iPC breaches with reference to past iPC breaches and anticipated iPC breaches in the future (see Kozlowski, 2009 for the connection between past, current, and future events). As such, a core assumption of the currently proposed model is that perceptions of iPC breach do not occur in a vacuum where past experiences are evaluated independently of the current events. Rather, employees are embedded within dynamic social systems and personal affective cycles that involve constant renegotiation of reciprocal relationships when the demands of the environment change. However, when these renegotiations occur, the history of how the iPCs has been negotiated in the past becomes salient. Specifically, this means that when a deviation from organizational obligations occurs, the events that were perceived as an iPC breach in the past will have an influence on whether or not the employee perceives the current deviation as surpassing their personal zone of acceptance, and thus as a new iPC breach (Schalk and Roe, 2007). Depending on the frequency of past iPC breaches, employees may be more likely to perceive a current deviation from an organizational obligation as an iPC breach. For example, the perception of more past iPC breaches may prime employees to expect future iPC breaches and decrease trust that the organization will not fail on their obligations again.

This perspective on the evolution of iPCs implies past experiences will have a strong influence on (1) the evaluation of whether a deviation from an organizational obligation has surpassed one’s personal tolerance or threshold and an iPC breach has occurred, (2) how employees will respond in relation to their specific workplace requirements and their own personal well-being, over time.

### Threshold Model of Ideological Psychological Contract Breach

Traditionally, scholars have studied the relationship between perceptions of PC breach and changes in attitudes and/or behaviors in a linear way such that as perceptions of PC breach increase, a change in attitudes and/or behavior of equal magnitude is assumed to occur. In doing so, we often consider and measure PC breach as a perception that comes from any deviation from fulfillment of organizational obligations and directly relate it to an attitudinal and/or behavioral outcome measure; we assume that because PC breach occurred, it will also impact the outcome. This approach groups all types of PC breach and reactions to PC breach into one homogenous category. In doing so, it is assumed that the magnitude of deviation is directly reflected in the magnitude of the reaction. This overly simplified assumption does not appropriately consider additional contextual factors such as the work environment, number of past PC breaches, type of PC being breached, and the value that the agreement holds to the employee in both their acknowledgment of PC breach and their reactions to PC breach. In other words, this static and linear logic model fails to represent the dynamic experience of the workplace environment and the multiple sources of information that employees have when cognitively evaluating a PC breach (see also Hansen and Griep, 2016).

To more adequately capture the dynamic nature of PC breach over time, researchers have proposed that perceptions and reactions to PC breach should be conceptualized as a threshold model, rather than a linear relationship. Being cognizant of the fact that some researchers have considered changes in PC relationships to be continuous, where the accumulation of iPC breaches would gradually results in changes to attitudes and behaviors (e.g., Ng et al., 2010; Vantilborgh et al., 2016; Griep and Vantilborgh, 2018), others have theoretically and empirically supported a threshold model (Schalk and Roe, 2007; Rigotti, 2009). Based on the diversity of events that may be tied to the ideology of the organization and be used in evaluation of iPC breach, we will argue that a threshold approach to iPC breach more adequately captures how employees behave in response to deviations from fulfillment.

Initially discussed by Guzzo et al. (1994) and by Morrison and Robinson (1997), these threshold models more adequately capture how employees perceive PC breaches in the workplace by proposing that employees filter all available PC-related information to determine if a PC breach occurred or not. Building on this initial conceptualization, Schalk and Roe (2007) argued that not all instances of incongruence between organizational obligations and delivered inducements will result in perceptions of PC breach. They argue that employees have a personal level of tolerance toward deviations from PC fulfillment of organizational obligations, termed the zone of acceptance. Deviations within this zone may not even be actively acknowledged by the employee and therefore are not perceived as a PC breach. However, when a deviation becomes too large or is related to a highly valued obligation, employees may perceive this deviation as surpassing their personal tolerance limit, which results in perceptions of PC breach (Schalk and Roe, 2007). Following these propositions, we argue that employees’ in iPCs are more susceptible to perceiving events as falling outside of their personal tolerance limit because the ideological nature of the PC makes it highly tied to aspects of their personal identity. For example, an employee who works for an organization that focuses on clean environmental practices, enters into an iPC related to the shared ideology of striving for a healthier environment. This employee personally values this goal and would likely label himself/herself as an environmentalist to some extent. As a result, this employee will be scanning a wide array of organizational activities that are related to fulfilling this goal and are especially attuned to deviations from fulfillment because these deviations may not only be a threat to the shared values and purpose, but are also interpreted as a personal threat to the employee’s identity.

In response to these theoretical arguments, Rigotti (2009) empirically tested these thresholds (i.e., events surpassing one’s personal tolerance limit) to examine if changes in attitudes in reaction to PC breach only occurred after a certain level of deviation from the organizational obligation was perceived. Using data from permanent and temporary German employees, Rigotti (2009) demonstrated drastic changes in feelings of violation, job satisfaction, and turnover intentions occurred after a certain level of PC breach perceptions were exceeded. Extending this finding to the iPC, we thus propose the following:

*Proposition 1:* If a deviation from an organizational iPC obligation exceeds an employee’s personal tolerance limit, the employee will perceive an iPC breach.

Although some studies have argued for, and explored, these thresholds (e.g., Schalk and Roe, 2007; Rigotti, 2009; Griep et al., 2016), no attention has been given to the dynamic nature of these thresholds and the mechanisms that define an employee’s personal zone of acceptance. An assumption of PC research is that the PC goes through a period, at least initially, of re-evaluation and change to reflect current expectations and obligations (Rousseau, 2001). Still, we have yet to understand the effect this may have on how PC thresholds operate over longer periods of time. As argued above, when a deviation of fulfillment of an organizational obligation exceeds an employee’s tolerance level, that employee is likely to perceive an iPC breach.

Keeping with the dynamic nature of the PC, we also argue that this tolerance level is not static and is highly susceptible to the effects of repeated iPC breaches over time; as the number of iPC breaches increases, employees may continue to adjust their expectations in response. Specifically, we argue that as perceptions of iPC breach accumulate, employees become more sensitive to deviations from iPC fulfillment. They may no longer trust their employer to fulfill its obligations (Rousseau, 1995; Robinson, 1996), and as a result scan their environment with more vigilance in an anticipation of future organizational failures to fulfill their obligations (Morrison and Robinson, 1997). As a result, employees’ tolerance limit will continue to decrease over time, resulting in an increasingly narrow zone of acceptance toward deviations from iPC fulfillment; increasingly mundane deviations from iPC fulfillment will be noticed and acted upon. In sum, this means that as the number of perceived iPC breaches increases over time, an employee’s personal tolerance limit will decrease, resulting in a smaller zone of acceptance. Moreover, as an employee’s personal tolerance limit decreases, the likelihood of evaluating a deviation as an iPC breach increases over time. Therefore, we propose the following:

*Proposition 2:* As the number of perceived iPC breaches increases over time, an employee’s personal tolerance limit decreases, resulting in a smaller zone of acceptance.

*Proposition 3:* As an employee’s personal tolerance limit decreases, the likelihood of evaluating a deviation from the fulfillment of an organizational obligation as an iPC breach increases over time.

This increased vigilance also has implications for the duration of the time period between subsequent perceptions of iPC breach. Specifically, once employees have responded to an iPC breach by changing their attitudes and/or behaviors, they will maintain this attitude and/or behavior (i.e., attitudinal or behavioral maintenance period) while simultaneously being highly attuned to potential sources of future iPC breaches. This largely comes from the idea that these employees are more sensitive to potentially future harmful events, especially when they perceive that the deviation is relevant to them (Öhman et al., 2001), as is the case with iPCs that have a strong connection to aspects of employees’ personal identity (Ashforth and Mael, 1989). Because their sensitivity to the workplace environment has increased, they are more likely to perceive more iPC breaches over time, resulting in an acceleration of the relationship between perceived iPC breach and a decrease in personal tolerance levels for organizational deviations from iPC fulfillment. Hence, we propose the following:

*Proposition 4:* As an employee’s personal tolerance limit decreases, the duration of the maintenance period between subsequent iPC breaches shortens over time; iPC breaches repeat with increased frequency over time.

### Ideological Psychological Contract Breach and Work Effort

Previous PC research has consistently supported the relationship between perceptions of PC breach and negative attitudinal and/or behavior reactions (for a meta-analysis, see Zhao et al., 2007; Bal et al., 2008). This relationship is often attributed to an employee’s desire to “rebalance” the scales after experiencing a disruption in the reciprocal exchange between themselves and their organization. Regardless of the specific attitudinal and/or behavioral change, PC breach has largely been viewed as a negative event that results in negative employee reactions. However, recent empirical findings have found that reactions to PC breach may not always be negative, particularly in the context of iPCs (Vantilborgh et al., 2014). Vantilborgh et al. (2014) investigated perceptions of iPC and relational PC breach among volunteers and found that workers increased their work effort in reaction to an iPC breach but decreased their work effort in reaction to a relational PC breach. The authors argue that the norm of altruism might be an important driver of reactions to iPC breach and will continue to devote considerable effort in an attempt to secure the desired ideology. Vantilborgh et al. (2014) argue that these workers are so invested in the ideology of their organization that they need to ensure that organizational goals are still met and the ideology is secured, even in the aftermath of an iPC breach. In a similar vein, Turnley et al. (2003) found that “regular” paid employees may not always decrease their work effort in response to organizational failings. In sum, these findings indicate that perceptions of iPC breach may not always lead to negative behavioral outcomes, such as reduced performance, but can trigger, at least in the short-term, an increase in work effort. Although the current empirical findings regarding an increase in work effort because of iPC breach are limited, they are suggestive of a unique and counterintuitive relationship within the PC framework and warrant further development and investigation.

The underlying motivation for these reactions can be found in the organizational identification literature where the congruence between the values of the employee and the values of the organization result is positive organizational attitudes such as increased organizational attachment and satisfaction as well as behaviors including in-role and extra-role performance (for a review, see Riketta, 2005). Specifically, employees who feel attached to their organization would likely want it to succeed in the long-term and may even engage in extra-role behaviors in the short-term if it was deemed necessary to maintaining the shared value. Personal identification with the organization is also in line with the theoretical tenants of Social Identity Theory (Tajfel and Turner, 1979, 1985) where individuals are committed to, and identify with, larger organizational causes, embracing them as a part of themselves (Ashforth and Mael, 1989; Hogg, 2016). When the shared identity of the employee and the organization becomes more salient, such as with an iPC breach that potentially poses a threat to one’s identity, employees become more motivated to reaffirm that identity (Tajfel and Turner, 1979). In doing so, employees engage in behaviors, including increased work effort, that aim to protect that identity and secure the shared valued cause (Olkkonen and Lipponen, 2006). As such, these employees find themselves compensating for their perceptions of unmet expectations on behalf of the organization and continue to push themselves to work harder to ensure that outcomes are still met, even if it comes at a personal cost (Thompson and Bunderson, 2003). We propose the following:

*Proposition 5:* Perceptions of iPC breach will trigger an increase in work effort in the short-term when organizational identification is high.

Although in the short-term, it may appear positive that employees are increasing their contributions to their organization, in the face of iPC breach, but this effect is likely short-lived. According to Job Demands-Resource Theory (JDR; Bakker and Demerouti, 2014), employees are unable to effectively maintain workplace behaviors, such as high work effort, if their demands are not balanced with the resources they have available. Because exerting more work effort is an increased demand imposed by the employee themselves in response to a decrease in resources from the organization (i.e., iPC breach), they experience higher demands than their resources allow them to cope with. Demerouti et al. (2001) found that such increases in demands relate to the experience of exhaustion, a component of burnout. Building on this, when employees have already perceived iPC breaches in the past and are expecting to perceive more iPC breaches in the future (in line with previous arguments and propositions in this paper), they can no longer continue to increase their work effort in response to a foreseeable future accumulation of iPC breaches. That is, we propose there is a depletion process where the extended experiences of iPC continue to exhaust the resources that the employee has available to respond to the event of iPC breach (Muraven and Baumeister, 2000). In their Ego Depletion Theory, Muraven and Baumeister (2000) argue that individuals have a limited personal resource pool, which gets depleted with each new experience of iPC breach. Because people need these personal resources to combat further depletion, engage in problem-solving coping behaviors, and maintain important work behaviors, an accumulation of iPC breach perceptions over time is likely to result in a situation in which the magnitude of work effort increase with each perception of iPC breach will decrease until the demands exceed the capabilities of the employee. At which point, we posit the existence of a tipping point at which these employees may become susceptible to burnout and no longer be able to continue to increase their work effort in response to an iPC breach. It is important to note that this tipping point is idiosyncratic and may thus differ from employee to employee depending on the point at which the demands placed upon the individual come to exceed their personal coping resources. We thus propose the following:

*Proposition 6:* When the workplace demands exceed the coping resources the employee has available, they will no longer increase their work effort in response to an iPC breach.

### Ideological Psychological Contract Breach, Work Effort, and Burnout

The experience of a PC breach and the events that follow can be stressful for employees and cause strain (Gakovic and Tetrick, 2003) because these employees may have lost confidence in the likelihood that organizational obligations are, and will continue to be, met in the future.

As the number of iPC breaches increases over time, employees are unable to still perceive their organization as supportive of their shared values or purpose and perceive a diminished sense of control over their ability to contribute to the ideology, which may further contribute to a diminished sense of well-being (Shore and Tetrick, 1994). Moreover, when employees are suspicious of the other’s motives, they may believe that the organization’s current iPC fulfillment is a cover for future potential iPC breaches (Karagonlar et al., 2016). As a result, even in circumstances of iPC fulfillment, employees may experience strain in anticipation for future iPC breaches. This experienced strain has been to found to be the result of a loss, or anticipated loss, to employment and personal resources (Hobfoll, 1989, 2002; Mayerl et al., 2016) and a decreased sense of control and predictability in the context of PCs (Shore and Tetrick, 1994).

These findings are in line with the theoretical tenets of JDR where an imbalance between demands placed on the employee and available resources to cope and maintain their behavior, results in work-related stress and subsequent negative outcomes such as sickness absenteeism and lowered job performance (Bakker and Demerouti, 2014). Building on the arguments pertaining to iPC breach and strain presented throughout this paper, we argue that an employee’s increase in work effort in the aftermath of an iPC breach results in such an imbalance; increased work effort results in increased job-related demands, while perceptions of iPC breach result in decreased perceptions of control and predictability (i.e., decreased resources). When this process accumulates over time (i.e., growing number of iPC breaches leading to further increase of strain over time), it creates an increasingly larger imbalance between the job demands placed on employees and the amount of available resources to cope with it, which will result in a further increase of strain over time. Keeping with these arguments, we propose the following:

*Proposition 7:* Employees who repeatedly increase their work effort in response to an iPC breach will experience an increased level of strain over time.

Over time, JDR assumes that this prolonged imbalance between increasing demands and decreasing resources can bridge experiences of increased strain to a more paramount and finite experience of burnout (Demerouti et al., 2001; Stansfeld and Candy, 2006; Jourdain and Chênevert, 2010; Alarcon, 2011; Melamed et al., 2011). Burnout is traditionally characterized by instances of emotional exhaustion, depersonalization or cynicism, and reduced sense of personal accomplishment or efficacy (Maslach, 1982; Maslach et al., 2001). The development of other measures has also lead to burnout being conceptualized by personal, work-related, and client-related burnout (Kristensen et al., 2005) and emotional and physical exhaustion (Pines and Aronson, 1988). Regardless, a highly related three-factor structure remains the primary approach (Platsidou and Daniilidou, 2016).

Despite its increased study among all types of work (Melamed et al., 2011; Van den Broeck et al., 2017), burnout remains a prevalent concern in ideology-infused organizations with research examining occupations such as healthcare (Jourdain and Chênevert, 2010; Hall et al., 2016), the military (Chambel and Oliveira-Cruz, 2010), and non-profit organizations (Kanter and Sherman, 2016). These examinations have consistently found that employees and volunteers continue to experience work-related stress and strain as the demands placed upon them frequently exceed their available resources, which ultimately increases their susceptibility to burnout, especially to experiences of emotional exhaustion (Lee and Ashforth, 1996). While this model primarily conceptualized burnout as an outcome, it may also develop in stages over time where experiences of emotional exhaustion influence more extensive burnout related behaviors later on (Leiter, 1991; Maslach et al., 2001). Emotional exhaustion is often considered the primary stage in the burnout model and largely reflects the prolonged impact of strain on the employee as a result of increased demands and decreased resources. Such a stepwise approach to the development of burnout aligns with AET (Weiss and Cropanzano, 1996), in which a perceived incongruence of demands and resources triggered by iPC breach leads to strain, which, over time leads to the development of emotional exhaustion (Demerouti et al., 2002).

Although emotional exhaustion often surfaces as the most apparent symptom of burnout, its presence is not a sufficient condition to characterize the tipping point of the full burnout syndrome (Maslach et al., 2001). Over time these experiences of emotional exhaustion, tend to trigger other behaviors that contribute to components of depersonalization and reduced feelings of effectiveness (Maslach et al., 2001). The unfolding nature of these behaviors also aligns with the nature of AET (Weiss and Cropanzano, 1996). Because emotional exhaustion further depletes the resources an employee has to effectively cope with stressors, such as iPC breaches, in the work environment (Leiter, 1991) as well as their ability to engage in emotion-regulation in high emotional labor jobs characteristic of ideological-infused organizations (Grandey, 2000; Brotheridge and Grandey, 2002), employees are more likely to engage in affect driven behaviors such as an emotional withdrawal from work (Weiss and Cropanzano, 1996).

Throughout this paper, we argued that the evolution of iPC breaches and employees’ work effort occur in an accelerated and upward fashion with a finite point where increases in effort on behalf of the employee can no longer occur. This finite point or tipping point is idiosyncratic and may thus differ from employee to employee depending on the point at which the demands placed upon the individual come to exceed their personal coping resources and the pent-up strain will result in the development of burnout. This similarly parallels the nature of the strain and burnout relationship defined through the JDR model (Bakker and Demerouti, 2014), where there is a limit to the imbalance between demands and resources tolerated by the employee. As experiences of strain begin to accumulate over time, they too reach a finite point where the employee can no longer cope with them and they reach a breaking point, more commonly conceptualized as burnout. We thus propose the following:

*Proposition 8:* In parallel to increases in employee work effort, when the workplace demands exceed the coping resources the employee has available, strain will result in emotional exhaustion which ultimately leads to the development of burnout.

## Discussion

Taken together, the presented propositions conceptualize a model that explains how, over time, perceptions of iPC breach can lead to experiences of strain and burnout and the mechanisms by which this occurs (i.e., increased work effort, decreased thresholds, increased likelihood of perceiving deviations from fulfillment as an iPC breach, decreased maintenance periods). Our conceptual model can be found illustrated in **Figure 1**.

As employees in ideologically infused organizations navigate reciprocal relationships with their employers, they put themselves at risk of experiencing burnout for the sake of maintaining the shared values and ideology of themselves and their organization (i.e., the “double-edged sword” of meaningful work). These values and ideology are held so deeply by the employee that not fighting for them to be upheld would be perceived as a threat to their personal identity, as defined by Social Identity Theory (Tajfel and Turner, 1979, 1985), and as such, can strongly influence their behavior. As a result, we argue that perceptions of iPC breach result in compensatory behaviors on behalf of the employee where they increase their work efforts in response to an organizational failure to fulfill the iPC. However, as perceptions of iPC breaches and subsequent increases in work effort accumulate over time, these employees begin to experience a breakdown in their ability to maintain this pattern because the level of available resources remains stable or decreases while their exerted work effort increases over time; thereby creating an imbalance between the demands placed on the employee and the resources to cope and maintain behavior (Bakker and Demerouti, 2014). As a consequence, we argue that perceptions of iPC breach change in response to the passing of time and the increase of strain. Specifically, employees’ personal tolerance limit for what is perceived as an iPC breach diminishes with each incidence of iPC breach. As a result of this lowered tolerance limit, employees are now considering deviations from organizational iPC obligations that were previously not evaluated by employees in relation to their iPC, as surpassing their personal tolerance limit, thus increasing the likelihood that an iPC breach will be perceived. When iPC breaches are perceived to be occurring more frequently, the need for increases in work effort also accelerate. However, these increases can only occur a finite number of times before employees are no longer able to further increase their work effort without reaching a breaking point at which the accumulated strain translates into burnout.

### Theoretical Contributions

Our novel conceptual model contributes to the organizational literature in three ways. First, this conceptual model responds to the current research agenda regarding the integration of time into PC research (for a research call see Hansen and Griep, 2016). Previous models and approaches to this area of inquiry have not adequately theorized the effect that accumulations of iPC breaches have on the employment relationship over time, particularly with a focus on long-term outcomes such as experiences of burnout. By conceptualizing the mechanisms that contribute to future perceptions of PC breach such as changes in personal tolerance levels toward deviations due to increased work effort and accumulated strain, as well as their relationship with long-term outcomes, we can address how iPCs come about and unfold over time.

Second, we contribute to the broader field of PC research by focusing on a previously understudied type of PC, the ideological PC. Although the inducements and outcomes of this type of reciprocal relationship have gained increased consideration in recent years (for examples, see Bingham et al., 2014; Vantilborgh et al., 2014), a conceptual framework to explain the empirical findings that deviate from traditional PC type research (i.e., transactional and relational) has not yet be proposed. By integrating the limited empirical findings on work effort in relation to iPC breach (Vantilborgh et al., 2014) with their potential consequences, we aim to demonstrate both the short-term and long-term consequences of iPC breaches and provide a framework for what iPCs and iPC breach entail for employees and their organizations.

Finally, by conceptualizing strain and burnout as a negative outcome of accumulated iPC breach perceptions, we provide a framework that explains why certain types of occupations and organizations are more susceptible to employees experiencing burnout and the mechanisms underlying this association. For example, organizations such as healthcare, education, and non-profits are primarily focused around an ideology and characterized by high emotional labor and personal investment. In this context, when employees personally invest in their work, they are also bolstering the ideology and expect the organization to reciprocate this investment in kind.

However, the strain of continuously putting forth highly valued efforts with a perceived lack of reciprocation can only be tolerated for so long. Several authors have indeed demonstrated that these organizations and occupations are highly prone to stress and burnout. For example, the percentage of nurses experiencing burnout ranges from 10 to 25% depending on their unit position (Ribeiro et al., 2014; Adriaenssens et al., 2015) with even higher prevalence (between 25 and 45%) when measures of emotional exhaustion, depersonalization, and personal accomplishment are considered independently (Cañadas-De la Fuente et al., 2015). In considering the mechanisms proposed in this model in relation to these employees’ workplace experiences, we can better understand the events leading up to burnout and propose ways to mitigate the negative effects. This could include identifying specific events that are perceived as an iPC breach, ensure realistic expectations about what the organization is able to provide to the employee, providing resources to help employees cope with the experience of iPC breach, and identifying ways to facilitate recovery from breach-related strain.

### Additional Consideration and Future Directions

Our current model has focused on iPC. This was largely done for two major reasons. First, to allow for a theoretical exploration of the counterintuitive response employees seem to have in response to iPC breach (i.e., increasing their work effort). Second, to provide a framework to explain the prevalence of employee strain and burnout in ideology-infused organizations through both a consideration of responses to iPC breach and the long-term consequences of such responses in terms of the development of burnout. Although the current model was developed with an emphasis on iPCs, there are elements of the model that may extend to PC research more generally. For example, our discussion of a threshold model of PC breach as well as changes to employee behavior during maintenance periods between PC breaches may also reflect how employees experience relational and transactional breaches over time. Future studies should consider similar mechanisms to examine how employee’s attitudes (e.g., job satisfaction, commitment) and behaviors (e.g., performance) change over time with the accumulation of relational and transactional PC breaches. Moreover, the current model does not consider how the accumulation of iPC breaches may change the nature of the PC to be more relational or transactional, and the effect this has on the potential accumulation of iPC breaches and employee reactions over time. Future studies should examine if the nature of the PC changes to emphasize more relational and transactional obligations as employees’ personal tolerance level toward deviations from iPC fulfillment decreases.

An additional aspect of the iPC that warrants further consideration is the operationalization of “the organization.” Traditionally, we have considered the organization or its representatives (e.g., HR department, management) as the entity held responsible for both the making and breaking of the contract (Rousseau, 1995; Conway and Briner, 2002). However, we believe this may be too simple of a conceptualization for the purpose of the iPC. We argue that as the PC is oriented around the ideology of an organization, the reciprocal relationship and the values promised to be upheld are navigated though organizational symbols, mission and values statements, and the anticipated expectations of what the industry represents. This means that the ideologies and values that the employee expects to be fulfilled are manifested at a larger organizational level, rather than embodied by an individual representative of the organization. This differs from our traditional considerations in that an individual may not have the same capacity to contract with an overarching ideology and use different actions or inactions of the organization to determine perceptions of iPC breach. Future research should consider exploring how employees in an iPC view the organization they are holding their iPC with and who and what they look for to determine fulfillment or breach of their iPC.

Future research could also consider the impact of accumulated iPC breaches on the perception of shared values and ideology between the employee and the organization. Employees who enter into an iPC with their organization often do so because they perceive a congruence between their own values and the values that the organization espouses. A perception of value congruence between an employee and their organization has important consequences for employment outcomes (Amos and Weathington, 2008; Howell et al., 2012). However, in line with our proposed model, it could be argued that the reciprocal relationship erodes over time when employees perceive multiple iPC breaches and increased anticipation of future iPC breaches over time, which in turn could erode the perceived value congruence. As a result, employees may be likely to experience more iPC breaches (Bocchino et al., 2003), which in turn may make them more susceptible to strain and burnout; thereby further accelerate the strain to burnout trajectory over time (Naus et al., 2007).

While we have focused on strain and burnout in relation to the experience of iPC breach over time, other outcome variables may follow a similar trajectory. For example, to maintain appropriate patient care in the healthcare industry, nurses and other healthcare professionals may begin to act outside of their role when perceiving an iPC breach by for example providing medical treatment that they are not authorized to, obtaining health resources illegally, and engaging in work arounds to attempt to maintain patient care. In doing so, they may actually undermine the organization and the ideology they were intending to uphold by risking the safety of patients rather than improving it. Future research should consider examining escalations of these behaviors and their outcomes for the organization.

Finally, future research should consider how employee’s respond toward the organization following the experience of burnout in relation to the PC framework. Previous research has found that employees may use a number of approaches to deal with the experience of PC breach such as increased voice, exit, and neglect, and decreased loyalty (Turnley and Feldman, 1999). Combining these findings with research conducted on employees’ responses to burnout (Drake and Yadama, 1996; Jourdain and Chênevert, 2010), we proposed that employees will likely express increased turnover intentions and ultimately leave the organization. Future considerations should examine this further as well as explore ways that organizations can facilitate recovery from burnout to prevent employee exit.

### Methodological Recommendations

#### Analytical Approaches

We propose that scholars use (1) segmented or spline regression analyses, (2) hurdle regression models, (3) zero-inflated Poisson regression models, (4) liability threshold models, or (5) catastrophe models. First, segmented or spline regression analyses test for a threshold by dividing a linear regression line into two linear regimes in the relationship between perceptions of iPC breach and outcomes (e.g., work effort, strain, burnout). This implies that the slope of the regression line can change (i.e., threshold or personal level of tolerance) for different ranges of the independent variable or the magnitude of iPC deviations. The values at which this change in the regression line happens are referred to as break-points, transition points, switch-points, knots, joint-points, or thresholds (Muggeo, 2003; Seber and Wild, 2003). In other words, once a certain value of the independent variable surpasses the personal tolerance level (i.e., the knot in the regression line), the direction of the regression line changes. Researchers can either *a priori* define this knot or they can estimate its value. The former can be done by visually inspecting the scatter plot of the relationship between perceptions of iPC breach and outcomes and looking for sudden changes in the cluster of points or by relying on prior research that has already demonstrated the value of this knot (Kunst et al., 1993). However, because interpreting the scatter plot is highly subjective and prone to researcher-bias and because we currently have no prior research that has demonstrated the value of these knots, we and Rigotti (2009) would like to advise researchers to use the non-linear regression function in SPSS to estimate the knot value. To estimate this value, a moderated regression analysis is needed that includes an intercept of iPC breach (β_0_), plus the direct effect of iPC breach on outcomes (β_1_^∗^ iPC breach), plus their interaction effect [(β_2_^∗^ (iPC breach-knot1) ^∗^ (iPC breach ≥ knot1)]. In this moderated regression analysis, “knot1” refers to the specific value within the range of the independent variable at which the slope of the regression line changes. When this parameter is significant, it indicates that there is indeed a significant value for the independent variable at which the regression line suddenly changes; demonstrating the existence of a threshold or personal level of tolerance.

Although segmented or spline regression analyses are by far the most common and most straightforward techniques used to estimate the proposed thresholds (see Muggeo, 2003; Seber and Wild, 2003; Rigotti, 2009), we will also briefly touch upon some more specialized techniques to model thresholds. First, the idea of *hurdle models* (Mullahy, 1986) is that one first needs to cross a “hurdle” before one can move on to the experience of events. In the first part of such hurdle models, the transition stage, one determines whether the accumulations of iPC breaches has surpassed one’s personal tolerance level (i.e., the demands placed upon the individual exceed the capabilities to deal with the accumulation in strain following the accumulation of iPC breaches). In the second part of such hurdle models, the event stage, scholars can model the intensity of burnout reactions once one has crossed the hurdle following the accumulation of iPC breaches. Similar to hurdle models, *zero-inflated Poisson models* (Lambert, 1992) can be used to estimate a zero-inflated (similar to the transition stage) and a Poisson (similar to the event stage) stage. Specifically, a zero-inflated Poisson model assumes that with probability *p* the only possible observation is 0 (i.e., no iPC breaches), whereas the probability 1 – *p* is a Poisson random variable. Applied to our iPC model, this implies that when one does not perceive the accumulation of iPC breaches to have surpassed one’s personal tolerance level, no burnout will emerge, but as soon as this accumulation is believed to have surpass the personal tolerance level, burnout may occur according to a Poisson distribution. Third, derived from genetics, the *liability-threshold model* (Sorensen et al., 1995) is a threshold model of binary outcomes (e.g., yes versus no) in which a large number of independent variables are used to predict an overall ‘liability’ score which can range from zero (no liability) to one (perfect liability). The observed outcome is determined by whether the latent score is smaller or larger than the threshold. Finally, mainly used in the field of risk management, *catastrophe modeling* (e.g., Grossi et al., 2005) is a technique that can prove to be very useful when studying the proposed thresholds and its consequences. A catastrophe model starts by characterizing the risk of a phenomena (e.g., what is the likelihood of an iPC breach?) and its associated impact on the individual (e.g., how impactful will the iPC breach be for the individual?). During this initial step, the model tries to understand the potential hazard of an iPC breach for the individual. Next, the model characterizes the vulnerability of the individual (e.g., How much coping resources does the individual has left?; What other demands are placed on this individual?). The objective of this step would be to determine the vulnerability or susceptibility to damage to the individual at risk. Finally, from this measure of vulnerability, the impact on the individual can be calculated (e.g., does the individual develop strain or emotional exhaustion?). Suppose that an individual experiences a number of iPC breaches, *E*_i_, which could negatively impact the individual. Each iPC breach has a probability of occurrence, *p*_i_, and associated impact or loss, *L*_i._ The number of iPC breaches are assumed to have a probability mass function defined as *P*(*E*_i_ occurs) = *p*_i_ and *P*(*E*_i_ does not occurs) = (1 -*p*_i_). If an iPC breach, *E*_i,_ does not occur, the impact or loss, *L*_i_, is zero and the threshold is not crossed. Hence the expected impact on the individual is zero and no increase in work effort or strain is expected. However, if an iPC breach, *E*_i,_ does occur, the associated expected impact on the individual can be calculated as *E*[*L*] = *p*_i_^∗^ *L*_i._ Because our model assumes that one needs to experience an accumulation in iPC breaches, resulting in repeated increases in work effort, before one crosses a threshold or tipping point into the development of emotional exhaustion and burnout, we need to calculate the exceedance probability as followed: *EP*(*L*_i_) = *P*(*L* > *L*_i_) = 1 - *P*(*L* ≤ *L*_i_). The resulting exceedance probability reflects the probability that the impact on an individual exceeds, for example, his/her ability to cope with the accumulation in strain resulting from a continued increase in work effort.

#### Research Design Requirements

Because several of the propositions made in this paper are highly idiosyncratic (e.g., the meaning of “finite” in proposition 6 could be different from individual to individual) and time-sensitive (i.e., most propositions require an accumulation to a certain threshold or tipping point), we need to make different methodological choices compared to those one would normally make in cross-sectional or semi-longitudinal studies. First, logically associated with time-sensitive propositions, methodological rigor should concern the developmental path (instead of the score) because the explicit aim is to capture how the proposed model evolves over time (not exists at a stable point in time), until it results in the development of burnout. This has important implications for the number of measurement waves one uses: the lower the number of measurement waves, the higher the chance of making erroneous conclusions vis-à-vis real change and evolution over time. Because the actual pattern of change is unknown *a priori* (i.e., there are no prior studies to inform us on how often one can increase one’s work effort following iPC breach or how long strain can accumulate before it results in burnout), we would recommend to an intensive longitudinal design (Collins, 2006) in which different measurement waves follow up quickly after one another over a relatively long time frame; a design commonly known as high density repeated measurements. In addition to using high density repeated measurements, we also advice researchers to move away from a traditional variable-centered approach to a person-centered approach. In traditional variable-centered approaches, results represent a synthesis (or averaged estimate) of the relationships observed in every individual from the sample under study, without systematically considering the possibility that these relationships may meaningfully differ in subgroups of participants (see the idiosyncratic nature of our propositions). In contrast, person-centered approaches strive to identify distinct profiles of employees (i.e., a typology; see Bailey, 1994; Bergman, 2000). Typologies, or taxonomies, represent classification systems designed to help categorize individuals more accurately into qualitatively and quantitatively distinct profiles (e.g., employees for whom a single increase in work effort following iPC breach may result in burnout vs employees for whom repeated increases in work effort are required to succeed the threshold and push them into burnout).

## Conclusion

By considering both the employee’s reaction to iPC breaches and their accumulated impact over time, we developed a conceptual model where the consideration of time is paramount to future investigations of how employees in ideology-infused organizations experience strain and become susceptible to burnout. This model contributes to our overall understand of PC breach and the role of ideology as well as proposes a new mechanism for understanding employee’s experiences in some of our most valued industries. We are hopeful that our novel conceptual model, along with the outlined methodological recommendations, will stimulate many original and exciting avenues of research.

## Author’s Note

SK is a Master’s student at the Department of Psychology, University of Calgary. Her primary research interests involve the study of psychological contracts with a special focus on ideology-infused organizations and the health and well-being outcomes of employees.

YG is Assistant Professor at the Department of Psychology, University of Calgary and a Faculty Member at the Division of Epidemiology, Stress Research Institute, Stockholm University. His primary research interests center on psychological contracts, negative behavior at work, commitment, health and well-being, and time-dynamics.

## Author Contributions

SJ visualized and wrote the original draft. SJ and YG contributed to the conceptualization, methodology, project administration, finding the resources, supervision, and writing, reviewing, and editing.

## Conflict of Interest Statement

The authors declare that the research was conducted in the absence of any commercial or financial relationships that could be construed as a potential conflict of interest.
